# Identifying driver genes involving gene dysregulated expression, tissue-specific expression and gene-gene network

**DOI:** 10.1186/s12920-019-0619-z

**Published:** 2019-12-30

**Authors:** Junrong Song, Wei Peng, Feng Wang, Jianxin Wang

**Affiliations:** 1 Faculty of Management and Economics/Faculty of Information Engineering and Automation/Technology Application Key Lab of Yunnan Province, Kunming University of Science and Technology, Kunming, Yunnan 650500 People’s Republic of China; 2 School of Information Science and Engineering, Central South University, Changsha, Hunan 410083 People’s Republic of China

**Keywords:** Driver genes, Dysregulated expression, Tissue-specific expression, Human functional interaction network, Variation frequency

## Abstract

**Background:**

Cancer as a kind of genomic alteration disease each year deprives many people’s life. The biggest challenge to overcome cancer is to identify driver genes that promote the cancer development from a huge amount of passenger mutations that have no effect on the selective growth advantage of cancer. In order to solve those problems, some researchers have started to focus on identification of driver genes by integrating networks with other biological information. However, more efforts should be needed to improve the prediction performance.

**Methods:**

Considering the facts that driver genes have impact on expression of their downstream genes, they likely interact with each other to form functional modules and those modules should tend to be expressed similarly in the same tissue. We proposed a novel model named by DyTidriver to identify driver genes through involving the gene dysregulated expression, tissue-specific expression and variation frequency into the human functional interaction network (e.g. human FIN).

**Results:**

This method was applied on 974 breast, 316 prostate and 230 lung cancer patients. The consequence shows our method outperformed other five existing methods in terms of Fscore, Precision and Recall values. The enrichment and cociter analysis illustrate DyTidriver can not only identifies the driver genes enriched in some significant pathways but also has the capability to figure out some unknown driver genes.

**Conclusion:**

The final results imply that driver genes are those that impact more dysregulated genes and express similarly in the same tissue.

## Background

Cancer as a kind of genomic alteration disease each year deprives many people’s life [1–3]. It is acknowledged that cancer arise is due to the accumulation of mutations in a subgroup of genes which conferring growth advantage, allowing uncontrolled proliferation and avoiding apoptosis [4, 5]. With the development of next-generation sequencing technology, several large-scale cancer projects have generated a large amount of cancer genomic data, such as The Cancer Genome Atlas (TCGA) [6], International Cancer Genome Consortium (ICGC) [7], which enable the detection of thousands of mutations. However, not all mutations contribute to the cancer initiation and progression. The mutations that are important to the cancer development and provide selective growth advantage are called driver mutations, the opposite is termed as the passenger mutations [8, 9]. Some researches show that the number of passenger mutations far beyond the number of driver mutations [9]. For example, from 11 cancer types, there are only 2 to 6 mutations have been regarded as the driver mutations among 200 somatic mutations which including missense, nonsense, silent, non-coding, splice-site, non-stop mutations, frameshift insertions and deletions (indels) and inframe indels [9–12]. Besides, those important alterations are not uniformly distributed across the genome and target to some specific genes associated with important cellular functions such as cell survival, cell fate etc. [4, 13–15]. For example, the well-known tumor suppressor TP53 participate in defense mechanisms against cancer and their inactivation by alteration can increase the selective growth advantage of the cell [16]. The alterations of ERBB2 [17] and KRAS [18] can lead to the acquisition of new properties that provide some selective growth advantage or spread to remote organs. Hence, the biggest challenge to overcome cancer is how to precisely discriminate those driver genes which harboring driver mutations and have the capability to promote cancer development from those irrelevant passenger genes [11]. This act is essential to understand the tumor biology and designing precision therapies [4, 19].

Traditional methods to identify cancer driver genes are based on the assumption that driver mutations confer a selective advantage to tumor growth and they occur more frequently than expected by random chance [20]. This kind of methods such as Mutsig [21] and MuSic [22] successfully pinpoints part of recurrence genes. However, in fact, only a small number of genes are altered in a high percentage of patient. Much larger number of genes are altered infrequently [11]. Besides, due to the heterogeneity of cancer, it is so hard to properly estimate the background mutation rate that many errors may be introduced [23].

A promising angle to identify cancer driver genes is based on network since it is acknowledged that cancer genes are more closely related with each other within a group to perform a certain function [24]. HotNet [25] and HotNet2 [26] apply a propagation process that diffuse the score of mutation frequency through the whole gene-gene interaction network and extract significantly mutated subnetworks to identify driver genes. NBS [27] detects driver genes by taking the strategies similar to HotNet. However, NBS detects mutated subnetworks of each patient and uses a consensus clustering framework to merge subnetworks across all patients. Unlike previous methods that use global network information, MUFFINN [28] prioritizes the cancer driver genes by measuring the impact from all neighbors of mutated genes in the functional network. Although these network-based methods mentioned above proposed a new focus on the interacting relationship of cancer driver genes, most of them identified cancer driver genes only consider the patient-gene mutation profiles and topology of networks. Besides, they are too much rely on the known network which may create some false positive data [23].

To overcome these limitations, some researchers focus on combining the cancer gene’s functional interactive relationship and other biological properties to improve the precision of detecting cancer driver genes. For example, DriverNet [29] identifies cancer driver genes by estimating their effect on mRNA expression. Inspired by the rationale that cancer driver genes may be determined by their impact on expressions of downstream genes, DriverNet firstly identifies the downstream genes (called outlying genes) with significantly differential expressions and then constructs a bi-graph where one side is mutated genes and the other side is outlying genes. It selects the driver genes that connect to the most nodes in the outlying gene side. Shi et al. [30] further improve DriverNet method by introducing diffusion process on the bi-graph. DawnRank [31] ranks potential cancer driver genes based on both their own expression difference and their impact on the overall differential expression of the downstream genes in the molecular interaction network. LNDriver [24] is also designed on the basis of bi-graph, while it incorporates the DNA length to filter mutated gene at the first step.

Above mentioned bi-graph-based methods to some degree improve the accuracy of identifying cancer driver genes by adding biology profiles to the gene itself. However, the reliability of network still needs to do further improvement since most of known networks are built based on either or mix of large scale of computational and experimental data. This may directly impact the efficiency and precision of detecting novel driver genes [23]. Hence, the fundamental problem is to establish one model that can improve the reliability of network so as to improve the power of prediction. To achieve this, some researchers consider to incorporate specific biological profiles to assign a weight for each interaction such as the impact of differential expression information [32]. However, seldom of them considered the facts that the majority of cancer genes interact with each other to form functional modules and those modules should tend to be expressed similarly in the same tissue. Ganegoda et.al [33] use the tissue-specific data to predict the new disease-gene associations by measuring the gene expression in disease related tissues and achieved higher performance. Besides, previous studies found genetic disorders tend to manifest only in a single or a few tissues for a given disease [34]. Motivated by these, we want to refine the gene functional interaction network by considering expression similarity between each pair of mutated genes in the cancer’s related one or two tissues. Moreover, from the previous research, it is known that cancer driver genes are more likely to be frequently mutated across a cohort of patients and also dysregulate downstream genes’ expression.

Based on the facts mentioned above, we proposed a model called DyTidriver to predict cancer driver genes by integrating dysregulated expression profiles, tissue-specific expression profiles, modularity of mutated genes and variation frequency into the gene functional interaction network. In DyTidriver, considering the fact that cancer driver genes are likely dysregulate downstream genes’ expression, mutated genes were firstly filtered according to their impact on the expression of downstream genes. After that, mutated genes’ interactive network was weighted by considering gene-gene co-expression in specific tissues of each query disease and the relationship between mutated genes. Because the majority of cancer driver genes interact with each other to form functional modules and those modules tend to be expressed similarly in the same tissue. Finally, with respect to the facts that driver genes are more likely to be frequently mutated across a cohort of patients and interact with each other to form functional modules, the mutated genes were ranked by summing up the weighted graph and multiplying itself variation frequency. We explored our method to detect cancer driver genes of lung cancer, breast cancer and prostate cancer. The result shows that our method significantly outperforms other five existing methods [28–31] in terms of Fscore, Precision and Recall. Besides, the cociter analysis illustrates our method can not only identify some well-known cancer driver genes but also detects the unknown cancer driver genes with high co-occurrence ratio in some publications. Furthermore, the identified cancer driver genes also enrich in some significant pathways and biological functions.

## Methods

**Fig. 1:**
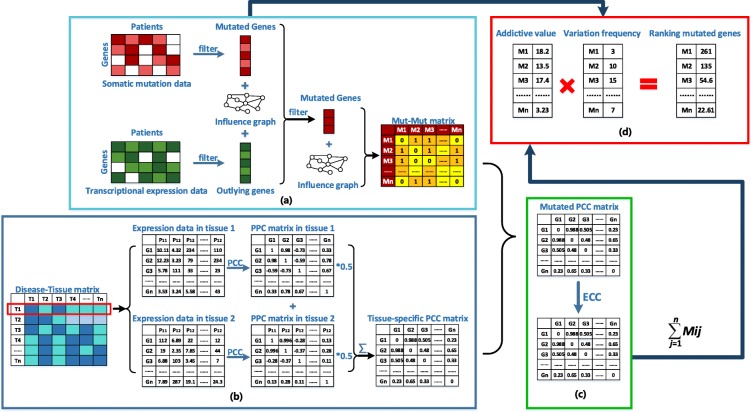
The workflow of Dytidriver. We divided our whole process of cancer driver gene identification into four steps and marked with ‘**a**’,’ **b**’, ‘**c**’, ‘**d**’. In the step ‘**a**’, we filtered the mutated genes for each patient according to whether or not it influenced the expression of downstream genes. Only the mutated genes which connect at least one outlying genes would be included in our study. Then, the filtered mutated genes for all patients were mapped to the human functional interaction network to construct the Mut-Mut matrix. The ‘**b**’ step is to generate the tissue-specific PCC matrix. For each cancer, we chose the top one or two tissues with the higher association score in disease-tissue matrix as the cancer related tissues such as the tissue 1 and tissue 2 for disease D1. For each tissue, we calculated its gene-gene pearson correlation values across the whole patients and then generated the gene-gene PCC matrix by keeping the absolute PCC values more than 0.3 while left setting to 0. If there are more than one tissue related to a cancer, the final tissue-specific PCC matrix is constructed by averaging the values in the gene-gene PCC matrix of each tissue. In the ‘**c**’ step, we constructed the ECC mutated matrix by utilizing the ECC equation. In the final ‘**d**’ step, we assigned each mutated gene in the network a score by summing up all the ECC values of its connecting edges and then multiply to its corresponding variation frequency. According to the scores, the mutated genes were ranked in a descending order and those ranked at the top list the were considered as potential driver genes

Our method consists of four steps (see Fig. 1). At first, we filtered the mutated genes for each patient according to whether or not it influenced the expression of downstream genes. Only the mutated genes that dysregualte downstream genes’ expression will be included in our study. Then, the remaining mutated genes for all patients were mapped to the human functional interaction network (human FIN) to construct the Mut-Mut matrix. Thirdly, the tissue-specific pearson correlation coefficient (PCC) matrix was constructed by calculating the co-expression values of mutated genes derived from downloaded tissue expression information after searching the disease-tissue matrix. Finally, we calculated the edge clustering coefficient (ECC) values for the interactions in the network which established at the last step and assigned each mutated gene in the network a score by firstly summing up ECC values of its connected edges and then multiplying the addictive result to its corresponding variation frequency. According to the scores, the mutated genes were ranked in a descending order and those ranked at the top of the list were considered as potential cancer driver genes.

### Experimental data

The datasets in this study derived from three places. The first part includes the somatic mutation data and their corresponding transcriptional expression data for each patient. Both of these datasets were downloaded from the TCGA website by utilizing the TCGA2STAT R packages. For our analysis, we focused on the somatic mutation and gene transcriptional expression data for 230 lung cancer patients, 974 breast cancer patients and 331 prostate cancer patients. The downloaded TCGA datasets include both tumor and normal patients: 58 of 230 lung, 110 of 974 breast and 52 of 331 prostate are normal patients.

The second part of dataset is the tissue-specific expression profiles. In order to find the most related tissues for each cancer type, we searched the tissue-disease matrix which can be downloaded from the reference [34]. Each entry in the matrix represents the covariance of a disease with a tissue through the way of counting the number of publications co-appearing the disease and tissue, relative to the number of publications mentioning the disease or tissue alone. It is acknowledged that genetic disorders tend to manifest only in a single or few tissues for a given disease [34]. Hence, we chose one or two of the most relevant tissues for each cancer type. Fortunately, the directly related tissue can be found for most of cancer type e.g. the lung tissue for lung cancer, prostate tissue for prostate cancer. However, we cannot find the breast tissue in the disease-tissue matrix. Instead, we chose the top two relevant tissues (e.g. prostate, ovary) with higher association score for breast cancer. In order to obtain the tissue-specific expression profiles, we used the Gene Expression Omnibus (GEO) database. Because GEO database is currently the largest and most famous expression data platform which stores relatively complete expression data. According to the identified most related tissues for each cancer type, we downloaded the gene expression details of each tissue sample from the GEO website by querying dataset GSE7307. The database lists the transcriptional profile of both normal and disease human tissues representing over 90 distinct tissue types by using the Affymetrix human U133 plus 2.0 array. At here, we used the R package called GEOquery to download the corresponding tissue expression information from the platform GPL570. The downloaded data is the expression profile matrix with genes and patients as the columns and rows respectively.

The last part of the dataset comes from the currently release version (2016) of human functional interaction network (human FIN) in which involving 12,275 genes and 46,0434 edges [35]. This network is constructed by extending curated pathways with non-curated sources of information, including protein-protein interactions, gene co-expression, protein domain interaction, Gene Ontology (GO) annotations and text-mined protein interactions, which cover close to 50% of the human proteome. The benchmarking of driver genes was downloaded from the NCG 4.0 which included 537 known cancer genes from the Cancer Gene Census [36] and 1463 candidate cancer genes that were derived from the manual curation of 77 whole genome or whole exome cancer-resequencing screenings [37] .

### Filtering mutated genes and constructing Mut-Mut matrix

The somatic mutation data were downloaded from TCGA website where records the information of mutated gene across patients. The genes that were mutated in at least one patient were kept and regarded as the mutated genes. Previous researches have pointed out that driver genes are more likely to regulate the expression of downstream genes [29–31]. Those gene whose expression were impacted significantly are called outlying genes. In order to acquire the outlying genes, we downloaded the transcriptional expression information from the TCGA website and calculated their z-scores. More specifically, for each gene and each patient, a gene was regarded as the outlying gene for the patient if its z-score > 2.0 or its z-score < − 2.0. The setting of threshold as ± 2.0 was referred to the DriverNet [29]. Then, we kept the mutated genes which have at least one connection with outlying genes in the human FIN while filtered out those having no connections with outlying genes. Finally, the remaining mutated genes were mapped to the human FIN to generated the binary Mut-Mut matrix in which the rows and columns are the remaining mutated genes and the element is 1 if there is a connection between the two mutated genes in the human FIN, 0 otherwise.

### Assigning weight to Mut-Mut matrix by PCC values

Since the majority of disease genes forming a common functional module tend to be expressed similarly in the same tissue and there exist too much false positive connections in the gene networks, in this work, we use tissue-specific expression profile to assign weights for the interactions of genes in order to improve the reliability of genes interactive network. For each cancer type, at first, we chose the most related tissue according to its association score in the disease-tissue matrix [34]. If there is at least one tissue related with a cancer in the disease-tissue matrix, its corresponding tissue expression information across a cohort of patients can be downloaded from the GEO website. After that, we calculated the gene-gene PCC values of downloaded tissue expression matrix across the whole patients and then generated the PCC matrix by keeping their absolute PCC values more than 0.3 while left setting to 0. The threshold setting was according to previous research [34]. At last, the average score of PCC matrix of each tissue was regarded as the final tissue-specific PCC matrix of the cancer type. We assigned a weight to values in the Mut-Mut matrix based on the tissue-specific PCC matrix. Specifically, if a mutated gene i connects to a mutated gene j in the Mut-Mut matrix (e.g. W(i,j) = 1), the PCC value of genes i and j was assigned to the corresponding entry of the Mut-Mut matrix otherwise the value was set to 0. Consequently, a weighted mutated PCC matrix denoted by W is constructed.

### Calculating the mutated gene score

Previous studies have found that cancer is the fact that genes act together in various signaling pathway and protein complexes [25]. Hence, in order to highlight the modularity of cancer driver genes, we calculated the ECC values for each pair of mutated genes in the mutated PCC matrix. The ECC value was normally used to measure the degree of closeness between two nodes in a network, which has been widely applied in detecting network modules [38–40]. We calculated the ECC values for each pair of mutated genes in the weighted mutated PCC matrix (denoted by Matrix W in Eq. 1). The higher ECC value means two genes are more likely to act together in a common module. The definition of ECC is as Eq. 1. After calculating the ECC score for each pair of mutated genes in the weighted mutated PCC matrix, we assigned each mutated gene a score (Mi) by summing up all ECC values of its connecting edges (see Eq. 2). It is known that cancer driver genes are more likely to be those frequently mutated in many patients. Hence, the final ranking score of each mutated gene was calculated by multiplying its variation frequency to its additive score (see Eq. 3). After that, all mutated genes were ranked in a descending order according to their ranking scores and the genes with the higher rank are more likely to be the cancer driver genes.

1 $$ ECC\left(i,j\right)=\frac{\sum_{k\in i\cap j}^n{W}_{ik}+{W}_{jk}}{\min \left({d}_i,{d}_j\right)} $$

2 $$ {M}_i=\sum \limits_{j\in {N}_i}^n ECC\left(i,j\right) $$

3 $$ {F}_i={V}_i\bullet {M}_i\kern0.5em $$

Where W denotes weighted mutated PCC matrix. *k* denotes the common neighbors between mutated gene i and gene j in the matrix W. W_ik_ is the weight between mutated gene i and gene k. *d*_*i*_ and *d*_*j*_ are the degrees of nodes i and j, respectively. Min (*d*_*i*_,*d*_*j*_) represents the maximal possible number of triangles that might include the edge(i,j). *N*_*i*_ is the set of all neighbors of mutated gene i. V_i_ denotes variation frequency of gene i which is measured by mutated times of gene i out of total patient counts.

### Statistic evaluation metrics

In order to evaluate the performance of our method, top N of ranked genes were selected as potential cancer driver genes. The accuracy of prediction depends on how well the predicted cancer driver genes match the real ones, which was measured by three widely used statistic metrics, Precision, Recall and Fscore.

$$ Precision=\frac{TP}{TP+ FP} $$

$$ Recall=\frac{TP}{TP+ FN} $$

$$ {F}_{score}=2\bullet \frac{Precision\bullet Recall}{Precision+ Recall} $$

where TP (true positive) is the number of predicted driver genes matched by known driver genes in benchmarking dataset. TN (true negative) is the number of not predicted driver genes that are not matched by known ones. FP (False Positive) is the number of predicted driver genes that are not matched by known driver genes. FN (false negative) is the number of known driver genes that are not matched by predicted ones.

### Enrichment analysis

Another evaluation metric is pathway and GO enrichment analysis in order to evaluate whether or not the predicted cancer driver genes share common biological functions. It is widely known that cancer is a disease of pathways and the somatic mutations target the cancer genes in a group of regulatory and signaling networks [25]. Besides, those cancer-related driver mutations recurrently occur in the functional regions of protein (such as kinase domains and binding domains) to interrupt the major biological functions [41]. In this study, we leveraged the DAVID database to do the KEGG pathway enrichment analysis and GO enrichment analysis [42].

## Results

In order to testify the effectiveness of our method, we applied our method and other four models:

DriverNet [29], DawnRank [31] and Diffusion algorithm [30], Muffinn [28] on the breast cancer, prostate cancer and lung cancer to identify their driver genes. Among them, the DriverNet, DawnRank and Shi’s Diffusion algorithm utilize the gene dysregulated expression information to identify outlying genes and construct the bipartite graph. These methods ranked mutated genes according to their connections with the outlying genes. The Muffinn method leverages both the variation frequency of mutated genes and the impact of their neighbors to design the ranking scores. It was further classified into two models: Muf_max and Muf_sum, according to considering the impact of either the most frequently mutated neighbor or all direct neighbors [28]. Unlike the DriverNet, DawnRank and Shi’s diffusion method that use gene dysregulated expression to construct bipartite graph, our study only employs the dysregulated expression profile to filter the mutated genes. Moreover, similar to the Muffinn method, we also consider the variation frequency of mutated genes and the impact of their direct neighbors. However, compared with other methods, our method not only integrates the features of dysregulated expression information, variation frequency and human FIN but also considers the modularity of mutated genes and their co-expression in the same tissue.

Running DawnRank demands expression data with normal and tumor samples. From the three cancer datasets, we can only download 110, 58, 52 tumor samples that have normal gene expression profiles for breast, lung and prostate respectively. Besides, we set the free parameter of DawnRank as three which was recommended by DawnRank authors [31].

### Comparing performance

**Fig. 2:**
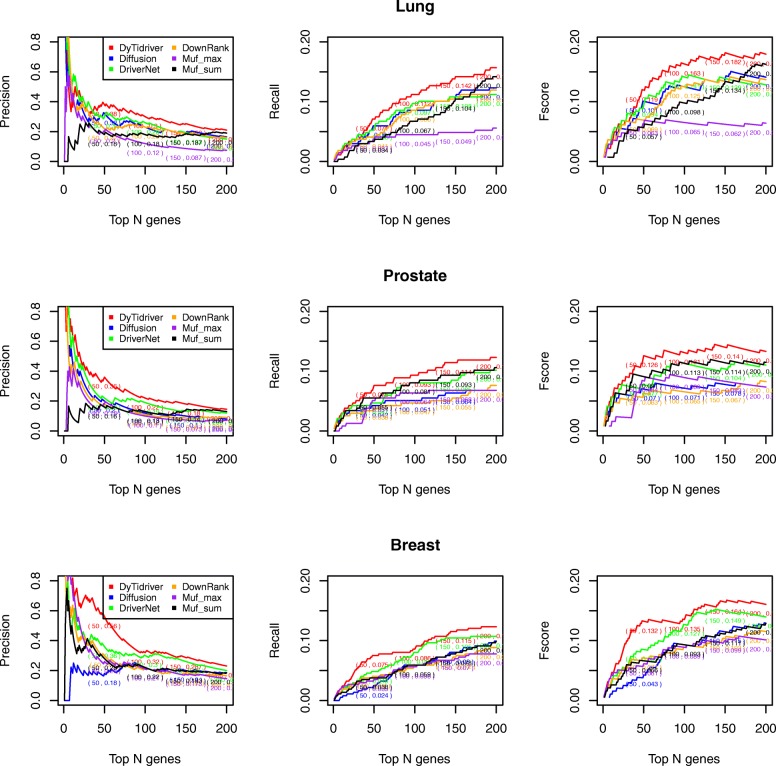
A comparison of the Precision, Recall, and Fscore for top ranking genes in the six methods. The X-axis represents the number of top-ranking genes. The Y-axis represents the score of the given metric

All the mutated genes were ranked in a descending order based on the scores assigned by each comparing method. After that, K of genes ranked in the top list were selected as candidate driver genes. According to the benchmark dataset, the Fscore, Recall, Precision values can be calculated to evaluate the performance of each method. With different values of K ranging from 1 to 200, the Fscore curve, Recall curve and Precision curve is drawn. The results are shown in the Fig. 2. In general, our results are superior to all of other four methods on the lung, prostate and breast cancer datasets. Compared with the other five methods, our model identifies the largest number of known drivers from NCG 4.0. For lung cancer, the Dytidriver and the other methods are tangled together when predicting small number of potential driver genes and then Dytidriver is significantly better than the other methods when the number of predicted driver genes increases from top 40 to 200. For prostate and breast cancer, our model demonstrated the best performance from beginning to the end. Similar to Muffinn, considering the variation frequency and the functional impact of direct neighbors, our method additionally takes advantage of the tissue-specific co-expression property and the modularity property which improve the precision of detecting driver genes to a higher level. Besides, the performance of Muf_max is worse than that of Muf_sum, which means it is inappropriate to judge a driver only based on the impact of single gene. DawnRank performed poorly among all comparing methods. The reason might be that only a limited number of cancer patients both have normal and tumor expression data for DawnRank.

### Enrichment analysis

We select the top 200 of cancer driver genes to do GO and pathway enrichment analysis. For lung cancer, in the biological process, the genes detected by our method enrich in the signal transduction, intracellular signaling cascade, transcription, metabolic process, regulation of cell death and apoptosis etc. In the cellular component, our results focus on the plasma membrane, organelle, cytoskeleton, lumen and cell fraction etc. In the molecular function, our results enrich in ion binding, nucleotide binding, ATP binding, transcription regulator activity etc. From the pathway aspect, our identified cancer driver genes enrich in some important cancer pathway, such as calcium signaling pathway, PI3K-Akt signaling pathway, mTOR signaling pathway.

With respect to the breast cancer, in biological process, our results enrich in the intracellular signaling cascade, signal transduction, regulation of transcription, metabolic process, regulation of cell death, phosphorylation, transcription, phosphorylation and cell proliferation. In the cellular component, our results enrich in the plasma membrane, organelle, lumen and cell fraction. In the molecular function, our results mainly enrich in the nucleotide binding, ATP binding, DNA binding, transcription regulator activity and kinase activity. In pathway analysis, our results enrich in Calcium signaling pathway, MAPK signaling pathway, PI3K signaling pathway, p53 signaling pathway etc.

In terms of prostate cancer, our results enrich in the regulation of transcription, signal transduction, adhesion molecules, regulation of GTPase activity etc. in biological process. For cellular component, our results enrich in nucleus, plasma membrane, cytosol, intracellular, protein complex etc. For molecular function, our results focus on protein binding, ATP binding, DNA binding, protein kinase activity and so on. From pathway aspect, our results enrich in the Calcium signaling pathway, PI3K signaling pathway, cAMP signaling pathway, mTOR signaling pathway.

### Cociter analysis

Because the benchmark cancer driver genes are incomplete, to further prove the prediction capability of our method in distinguishing potentially cancer driver genes, we adopted the literature mining method to figure out the co-citation times of the predicted driver genes with the keywords ‘cancer type’(i.e. breast, prostate or lung), ‘driver’ and ‘cancer’ in the cociter website [25]. The larger the number of times the gene co-appeared with the keywords, the stronger associations between them. In this study, Tables 1, 2 and 3 show the cociter analysis of top 30 of genes identified by our method for each cancer type. In order to illustrate the capability of our method to prioritize significant well-known cancer driver genes, we also listed genes ranking position in other five methods.

**Table 1:** Cociter analysis of top 30 lung cancer driver genes identified by our method

Genes	Cancer	Lung	Driver	Is_driver	DyTidriver	Diffusion	DriverNet	DawnRank	Muf_max	Muf_sum
TP53	6772	999	110	1	1	20	1	1	5	6
ZNF536	4	0	1	1	2	5015	NA	2689	849	79
EGFR	4748	2849	166	1	3	1	3	4	7	26
TSHZ3	4	1	1	0	4	2748	1295	2463	1268	188
PRUNE2	12	1	1	0	5	5211	NA	2623	2018	332
RYR2	4	3	2	0	6	757	20	558	128	25
SPTA1	3	2	1	0	7	221	6	15	12	36
ATP10D	1	0	0	0	8	1836	NA	2825	2667	873
ANKIB1	2	1	0	0	9	1607	NA	2572	4107	2080
ZNF521	2	0	1	1	10	5025	NA	3058	1906	302
NES	192	31	5	0	11	1483	NA	1461	3094	1138
PIK3CA	1199	183	54	1	12	2	5	112	430	81
TLR4	417	591	9	1	13	71	45	3	672	138
NF1	165	16	11	1	14	34	56	21	389	139
FAT4	45	7	2	0	15	3106	839	1961	970	119
ASH1L	4	1	1	0	16	1506	NA	2289	2549	761
PRKCB	41	11	1	1	17	5	12	NA	442	92
SLC12A1	2	2	1	0	18	1647	NA	3038	4006	1750
CTNNB1	2517	340	44	1	19	6	21	NA	51	27
PLCB1	9	7	1	0	20	25	22	27	745	91
APOB	27	4	2	0	21	117	7	8	664	42
MET	1045	348	40	0	22	21	37	7	427	186
GRIN2B	13	3	2	0	23	18	39	120	397	135
UBC	134	17	2	0	24	3	4	NA	137	1
SASH1	13	3	1	0	25	1537	NA	1325	5100	3080
HGF	393	174	7	0	26	47	84	40	398	1192
BRAF	2175	270	126	1	27	70	75	155	392	150
UBA6	1	1	1	0	28	5263	NA	NA	2957	980
PTPRZ1	12	1	1	0	29	3366	NA	2402	894	289
TAF1L	2	1	1	0	30	557	57	547	10	130

The second to the fourth column show the co-appeared times of top 30 identified genes with ‘driver’, ‘lung’ and ‘cancer’ (from the left to the right). Is_Driver indicates whether the given gene is a driver gene or not in the benchmark dataset. The left columns represent the ranking positions of identified genes in Dytidriver, Diffusion, DriverNet, DawnRank, Muf_max, Muf_sum respectively

**Table 2:** Cociter analysis of top 30 prostate cancer driver genes identified by our method

Genes	Cancer	Prostate	Driver	is driver	DyTidriver	Diffusion	DriverNet	DawnRank	Muf max	Muf sum
TP53	6772	298	110	1	1	1	1	1	38	4
CTNNB1	2517	170	44	1	2	2	2	21	40	9
ASH1L	4	0	1	0	3	1703	NA	NA	653	78
SPOP	43	24	4	1	4	1721	3	169	8	3
ATM	1377	61	5	0	5	13	11	12	36	14
PTEN	3047	642	64	1	6	700	94	NA	39	37
TTN	10	0	2	0	7	1724	22	14	2	2
FOXA1	182	69	10	0	8	17	5	3	37	10
KMT2D	25	2	2	0	9	855	54	NA	NA	NA
PIK3CA	1199	34	54	1	10	7	10	NA	282	36
DYNC1H1	9	1	2	0	11	66	19	51	219	72
CDH12	4	0	0	0	12	1511	NA	755	349	296
BRAF	2175	33	126	1	13	326	63	36	348	34
AKT1	2152	317	23	1	14	20	23	NA	52	33
FAT3	1	1	1	0	15	19	26	75	NA	NA
LRP4	7	0	2	0	16	1440	NA	NA	1426	541
GRIN2B	13	0	2	0	17	74	33	NA	220	90
KMT2C	23	2	4	0	18	613	27	NA	NA	NA
NCOR1	109	27	3	1	19	59	77	58	41	60
HSPA8	96	9	1	0	20	10	8	NA	438	67
OBSCN	7	0	0	0	21	1714	168	408	1	24
GRIN2A	5	0	1	0	22	285	92	85	374	73
PCDHA12	1	0	0	0	23	1453	271	197	324	65
MED12	19	4	4	0	24	376	162	157	317	84
STAT3	1824	147	27	0	25	16	15	5	58	8
PCDH18	2	1	1	0	26	1656	93	66	262	39
CDH23	5	0	1	0	27	457	97	NA	295	63
SPTA1	3	0	1	0	28	1719	16	9	221	15
UFL1	7	0	1	0	29	NA	NA	NA	1238	1265
SP1	393	38	3	1	30	8	9	NA	86	5

The second to the fourth column show the co-appeared times of top 30 identified genes with ‘driver’,‘prostate’ and ‘cancer’ (from the left to the right). Is_driver indicates whether the given gene is a driver or not in benchmark dataset. The left columns represent the ranking positions of identified genes in Dytidriver, Diffusion, DriverNet, DawnRank, Muf_max, Muf_sum respectively

**Table 3:** Co-citer analysis of top 30 breast cancer driver genes identified by our method

Genes	Cancer	Breast	Driver	is driver	DyTidriver	Diffusion	DriverNet	DawnRank	Muf max	Muf sum
TP53	6772	1356	110	1	1	233	1	2	7	2
PIK3CA	1199	334	54	1	2	156	2	1	2	3
MAP 3 K1	135	62	2	1	3	128	18	4	899	28
GATA3	154	122	8	1	4	85	13	6	888	17
CDH1	1410	358	19	1	5	42	4	10	1	6
ERBB2	5335	4332	78	1	6	72	64	90	8	73
UBC	134	30	2	0	7	240	3	122	22	1
NCOR1	109	45	3	1	8	139	12	48	6	68
ASH1L	4	0	1	0	9	1097	NA	1986	1846	729
PIK3R1	131	21	7	1	10	160	10	26	13	45
EP300	269	86	4	1	11	68	5	178	367	4
DYNC1H1	9	2	2	0	12	63	8	17	1017	107
HUWE1	29	4	3	0	13	251	28	45	9	112
PTEN	3047	672	64	1	14	185	98	193	3	79
MAP 3 K13	2	0	1	1	15	6189	NA	3303	2654	2045
NF1	165	24	11	1	16	141	41	19	4	144
TTN	10	1	2	0	17	2581	6	5	717	5
TPP2	4	0	2	0	18	1041	NA	2674	3172	2926
UFL1	7	1	1	0	19	802	NA	NA	3493	3129
BRCA1	4652	4017	22	1	20	25	11	NA	361	27
BACH2	8	1	2	0	21	810	1182	2366	2298	1079
JAK2	382	92	19	1	22	118	32	NA	73	119
ERBB3	354	178	4	1	23	73	29	8	10	207
ERBB4	350	220	4	1	24	74	56	276	18	410
MAP 2 K4	70	10	2	1	25	127	34	23	898	86
CTCF	63	21	3	1	26	55	20	211	1027	29
PRKCB	41	9	1	1	27	174	59	31	80	151
SASH1	13	8	1	0	28	1011	NA	NA	3706	4179
TAF1	10	3	1	1	29	225	86	33	359	19
SPTA1	3	0	1	0	30	212	17	25	1018	109

The second to the fourth column show the co-appeared times of top 30 identified genes with ‘driver’, ‘breast’ and ‘cancer’ (from the left to the right). is_driver indicates whether the given gene is a driver or not in the benchmark dataset. The left columns represent the ranking positions of identified genes in Dytidriver, Diffusion, DriverNet, DawnRank, Muf_max, Muf_sum respectively

For lung cancer, Table 1 shows some well-studied cancer driver genes were ranked in the top 30 by our methods, but were put in the latter positions by other methods. For example, Phosphatidylinositol 3-kinases (PI3Ks) are well known regulators of cellular growth and proliferation. It was ranked 12th by our method while ranked 112th by Dawnrank, 430th by Muf_max, 81th by Muf_sum. Toll-like receptor-4 (TLR4) in human tumors often correlates with chemoresistance and metastasis [43] which was ranked 13th by our method, ranked 71th by Diffusion algorithm while ranked 672th by Muf_max and 138th by Muf_sum. The oncogenic BRAF(V600E) mutation results in an active structural conformation characterized by greatly elevated ERK activity [44]. It was identified as the known cancer driver genes but ranked 70th, 75th, 155th, 392th and 150th by Diffusion, DriverNet and DawnRank, Muf_max and Muf_sum respectively. Our method can not only prioritize the significant cancer driver genes but also identify some potential cancer driver genes which were neglected by the NCG 4.0 such as the NES, MET and HGF. Especially for the MET, some researchers found that high MET gene copy number leads to shorter survival in patients with non-small cell lung cancer. MET co-existed with key words, ‘cancer’, ‘lung’ and ‘driver’ for 1045, 348 and 40 times.

For the prostate cancer as shown in Table 2, our method also identified some high-ranking significant driver genes, including TP53, CTNNB1, PTEN, PIK3CA and so on. What we want to mention is the famous tumor suppressor PTEN which is frequently inactivated in human prostate cancer [45]. It was ranked 6th by our method but strangely put in the 700th by Diffusion algorithm, 94th by DriverNet and even neglected by DawnRank. Furthermore, the results show DawnRank missed more than one significant cancer driver genes including PTEN, PIK3CA and AKT1. BRAF which involves in prostate related RAS/RAF/ERK signaling pathway [28] was ranked 13th by our methods while 326th by Diffusion algorithm, 63th by DriverNet, 36th by DawnRank, 348th by Muf_max and 34th by Muf_sum. Besides, some high associated genes ignored by NCG 4.0 are also ranked in the top list of our method. The ATM (ataxia telangiectasia mutated) kinase plays an essential role in maintaining genome integrity by coordinating cell cycle arrest, apoptosis, and DNA damage repair [46]. It was missed by the NCG 4.0 but co-appeared with ‘cancer’ for 1377 times, with ‘prostate’ for 61 times and with ‘driver’ for 5 times. Forkhead box protein A1 (FOXA1) modulates the transactivation of steroid hormone receptors and thus may influences tumor growth and hormone responsiveness in prostate cancer [47]. It was ranked 8th by our method while neglected by NCG 4.0. In addition, the transcription factors SP1 also has been missed by NCG 4.0.

For breast cancer in Table 3, our method successfully achieved a high precision in identifying the top 10 cancer driver genes with 8 out of 10 accuracy rates. The well-studied breast cancer driver genes including TP53, PIK3CA, MAP 3 K1, CDH1, ERBB2 and PTEN were also put in the top list of our method. Among those known breast cancer driver genes, the top three cancer driver genes (TP53, PIK3CA, MAP 3 K1) identified by our methods were ranked 233th, 156th and 128th respectively by Diffusion algorithm. The HER2 (official name is ERBB2) gene encodes a membrane receptor in the epidermal growth factor receptor family amplified and over expressed in adenocarcinoma [48]. It was regarded as the important cancer driver gene by many researchers and ranked 6th by our method while 72th, 64th, 90th, 73th by Diffusion algorithm, DriverNet, DawnRank and Muf_sum respectively. The breast cancer suppressor gene PTEN was ranked 14th by our method while 185th, 98th, 93th and 79th by Diffusion, DriverNet, DawnRank and Muf_sum receptively. Besides, the BRCA1 and JAK2 that co-cited with ‘cancer’ and ‘breast’ for many times were also missed by the DawnRank.

## Discussion

The core step to overcome cancer is to identify the cancer driver genes which can promote cancer evolvement and development. However, it is a hard task since cancer is heterogeneous and there are too much irrelevant passenger genes. Recently, many methods try to shorten the distance to the truth. However, these methods still have some limitations. For example, they ignored many driver genes with low variation frequency and highly depend on the error-prone network. Inspired by the fact that cancer genes forming functional modules tend to be expressed similarly in the same tissue, we considered to improve the reliability of the gene functional interaction network by incorporating the expression similarity between mutated gene pairs in the cancers’ related tissues. In order to obtain the tissue-specific expression profiles, we used the GEO database. Because GEO database is currently the largest and most famous expression data platform which stores relatively complete expression data. The GEO dataset which we used in this work was consisted of a total of 677 patients, including cancer and normal patients, covered over 90 distinct tissue types and was created by the same organization using the same experimental technology. Although our model is superior to the other methods, it still has some limitations. For example, the datasets used in this work come from different projects: TCGA and GEO. Although, we just use the GEO dataset to calculate the co-expression values of mutated genes in a specific tissue. The likelihood is that there exists ambiguous since the heterogeneous within different patients. Therefore, in order to release this concern, in the future, we consider to unify the dataset as far as possible.

## Conclusion

In this work, we proposed a new method to identify cancer driver genes by integrating the gene dysregulated expression, tissue-specific expression and variation frequency into the functional interaction network. Compared to other network-based methods, our method not only considered that driver genes have impact on the expression of downstream genes, but also took advantage of the modularity property of driver genes, their co-expression in specific tissues and itself variation frequency. We compared our results with other four similar methods and did cociter analysis and enrichment analysis. From the results, we can easily draw the conclusion that our method has the capability to identify the cancer driver genes with high precision and meanwhile detect some potential unknown cancer driver genes. Besides, the enrichment analysis also illustrates that the top ranking cancer driver genes in our list enrich in some significant cancer-related pathways and implement important functions [48].

## Data Availability

The source code and datasets used in this research can be downloaded from https://github.com/weiba/DyTidriver.

## Abbreviations

ECC: edge clustering coefficient
GEO: Gene Expression Omnibus
human FIN: human functional interaction network
ICGC: Cancer Genome Consortium
PCC: Pearson correlation coefficient
TCGA: The Cancer Genome Atlas

## References

[1] Baker SG (2017). The questionable premises underlying the search for cancer driver mutations and cancer susceptibility genes. Organisms Journal of Biological Sciences.

[2] Kumar S, Warrel J, Mcgillivray P, Meyerson W, Li S, Salichos L, et al. Passenger mutation landscape in cancer genomes. AACR. 2018:1279–9.

[3] Yates LR, Campbell PJ (2012). Evolution of the cancer genome. Nat Rev Genet.

[4] Collier O, Stoven V, Vert J-P (2019). LOTUS: a single-and multitask machine learning algorithm for the prediction of cancer driver genes. PLoS Comput Biol.

[5] Greenman C, Stephens P, Smith R, Dalgliesh GL, Hunter C, Bignell G (2007). Patterns of somatic mutation in human cancer genomes. Nature..

[6] Network CGAR (2008). Comprehensive genomic characterization defines human glioblastoma genes and core pathways. Nature..

[7] Consortium ICG (2010). International network of cancer genome projects. Nature..

[8] Stratton MR, Campbell PJ, Futreal PA (2009). The cancer genome. Nature..

[9] Pon JR, Marra MA (2015). Driver and passenger mutations in cancer. Annual Review of Pathology: Mechanisms of Disease.

[10] Kandoth C, McLellan MD, Vandin F, Ye K, Niu B, Lu C (2013). Mutational landscape and significance across 12 major cancer types. Nature..

[11] Vogelstein B, Papadopoulos N, Velculescu VE, Zhou S, Diaz LA, Kinzler KW (2013). Cancer genome landscapes. Science.

[12] Network CGAR (2017). Integrated genomic and molecular characterization of cervical cancer. Nature..

[13] Ding L, Getz G, Wheeler DA, Mardis ER, McLellan MD, Cibulskis K (2008). Somatic mutations affect key pathways in lung adenocarcinoma. Nature..

[14] Morin RD, Mendez-Lago M, Mungall AJ, Goya R, Mungall KL, Corbett RD (2011). Frequent mutation of histone-modifying genes in non-Hodgkin lymphoma. Nature..

[15] Paez JG, Jänne PA, Lee JC, Tracy S, Greulich H, Gabriel S (2004). EGFR mutations in lung cancer: correlation with clinical response to gefitinib therapy. Science..

[16] Chen P-L, Chen Y, Bookstein R, Lee W-H (1990). Genetic mechanisms of tumor suppression by the human p53 gene. Science..

[17] Gemignani ML, Schlaerth AC, Faina B, Barakat RR, Oscar L, Robert S (2003). Role of KRAS and BRAF gene mutations in mucinous ovarian carcinoma. Gynecol Oncol.

[18] Schechter AL, Stern DF, Vaidyanathan L, Decker SJ, Drebin JA, Greene MI (1984). The neu oncogene: an erb-B-related gene encoding a 185,000-Mr tumour antigen. Nature..

[19] Reimand J. Candidate non-coding driver mutations in super-enhancers and long-range chromatin interaction networks across 1,800 whole cancer genomes. AACR. 2018:2354–4.

[20] Lawrence MS, Stojanov P, Polak P, Kryukov GV, Cibulskis K, Sivachenko A (2013). Mutational heterogeneity in cancer and the search for new cancer-associated genes. Nature..

[21] Banerji S, Cibulskis K, Rangel-Escareno C, Brown KK, Carter SL, Frederick AM (2012). Sequence analysis of mutations and translocations across breast cancer subtypes. Nature..

[22] Dees ND, Zhang Q, Kandoth C, Wendl MC, Schierding W, Koboldt DC (2012). MuSiC: identifying mutational significance in cancer genomes. Genome Res.

[23] Cheng F, Zhao J, Zhao Z (2015). Advances in computational approaches for prioritizing driver mutations and significantly mutated genes in cancer genomes. Brief Bioinform.

[24] Wei P-J, Zhang D, Xia J, Zheng C-H (2016). LNDriver: identifying driver genes by integrating mutation and expression data based on gene-gene interaction network. BMC bioinformatics.

[25] Vandin F, Upfal E, Raphael BJ (2011). Algorithms for detecting significantly mutated pathways in cancer. J Comput Biol.

[26] Leiserson MD, Vandin F, Wu H-T, Dobson JR, Eldridge JV, Thomas JL (2015). Pan-cancer network analysis identifies combinations of rare somatic mutations across pathways and protein complexes. Nat Genet.

[27] Hofree M, Shen JP, Carter H, Gross A, Ideker T (2013). Network-based stratification of tumor mutations. Nat Methods.

[28] Cho A, Shim JE, Kim E, Supek F, Lehner B, Lee I (2016). MUFFINN: cancer gene discovery via network analysis of somatic mutation data. Genome Biol.

[29] Bashashati A, Haffari G, Ding J, Ha G, Lui K, Rosner J (2012). DriverNet: uncovering the impact of somatic driver mutations on transcriptional networks in cancer. Genome Biol.

[30] Shi K, Gao L, Wang B (2016). Discovering potential cancer driver genes by an integrated network-based approach. Mol BioSyst.

[31] Hou JP, Ma J (2014). DawnRank: discovering personalized driver genes in cancer. Genome medicine.

[32] Guo W-F, Zhang S-W, Liu L-L, Liu F, Shi Q-Q, Zhang L (2018). Discovering personalized driver mutation profiles of single samples in cancer by network control strategy. Bioinformatics..

[33] Ganegoda GU, Wang J, Wu F-X, Li M (2014). Prediction of disease genes using tissue-specified gene-gene network. BMC Syst Biol.

[34] Lage K, Hansen NT, Karlberg EO, Eklund AC, Roque FS, Donahoe PK (2008). A large-scale analysis of tissue-specific pathology and gene expression of human disease genes and complexes. Proc Natl Acad Sci.

[35] Wu G, Feng X, Stein L (2010). A human functional protein interaction network and its application to cancer data analysis. Genome Biol.

[36] Futreal PA, Coin L, Marshall M, Down T, Hubbard T, Wooster R (2004). A census of human cancer genes. Nat Rev Cancer.

[37] An O, Pendino V, D’Antonio M, Ratti E, Gentilini M, Ciccarelli FD: NCG 4.0: the network of cancer genes in the era of massive mutational screenings of cancer genomes. *Database*. 2014;2014. 10.1093/database/bau015 PMC3948431 24608173

[38] Wang J, Li M, Chen J, Pan Y (2011). A fast hierarchical clustering algorithm for functional modules discovery in protein interaction networks. IEEE/ACM Transactions on Computational Biology and Bioinformatics (TCBB).

[39] Peng W, Li M, Chen L, Wang L (2017). Predicting protein functions by using unbalanced random walk algorithm on three biological networks. IEEE/ACM transactions on computational biology and bioinformatics.

[40] Peng W, Wang J, Zhao B, Wang L (2015). Identification of protein complexes using weighted PageRank-nibble algorithm and core-attachment structure. IEEE/ACM Transactions on Computational Biology and Bioinformatics (TCBB).

[41] Song J, Peng W, Wang F: An Entropy-based method for identifying mutual exclusive driver genes in cancer. *IEEE/ACM transactions on computational biology and bioinformatics*. 2019.2.7;10.1109/TCBB. 10.1109/TCBB.2019.2897931 30763245

[42] Huang DW, Sherman BT, Lempicki RA (2008). Systematic and integrative analysis of large gene lists using DAVID bioinformatics resources. Nat Protoc.

[43] Rajput S, Volk-Draper LD, Ran S (2013). TLR4 is a novel determinant of the response to paclitaxel in breast cancer. Mol Cancer Ther.

[44] Lee MH, Lee SE, Kim DW, Ryu MJ, Kim SJ, Kim SJ (2011). Mitochondrial localization and regulation of BRAFV600E in thyroid cancer: a clinically used RAF inhibitor is unable to block the mitochondrial activities of BRAFV600E. The Journal of Clinical Endocrinology & Metabolism.

[45] Kwak MK, Johnson DT, Zhu C, Lee SH, Ye D-W, Luong R (2013). Conditional deletion of the Pten gene in the mouse prostate induces prostatic intraepithelial neoplasms at early ages but a slow progression to prostate tumors. PLoS One.

[46] Fan C, Quan R, Feng X, Gillis A, He L, Matsumoto ED (2006). ATM activation is accompanied with earlier stages of prostate tumorigenesis. Biochimica et Biophysica Acta (BBA)-Molecular Cell Research.

[47] Gerhardt J, Montani M, Wild P, Beer M, Huber F, Hermanns T (2012). FOXA1 promotes tumor progression in prostate cancer and represents a novel hallmark of castration-resistant prostate cancer. Am J Pathol.

[48] Wen W, Xiao N, Bender R, Ghazalpour A, Tan Z, Swensen J (2015). Mutations in the kinase domain of the HER2/ERBB2 gene identified in a wide variety of human cancers. The Journal of Molecular Diagnostics.

